# Malignant perivascular epithelioid cell tumor mimicking jugular foramen schwannoma: A case report and literature review

**DOI:** 10.1016/j.heliyon.2020.e03200

**Published:** 2020-01-17

**Authors:** Noritaka Komune, Shogo Masuda, Ryuji Yasumatsu, Takahiro Hongo, Rina Jiromaru, Satoshi Matsuo, Osamu Akiyama, Nana Tsuchihashi, Nozomu Matsumoto, Hidetaka Yamamoto, Takashi Nakagawa

**Affiliations:** a Department of Otorhinolaryngology, Graduate School of Medical Sciences, Kyushu University, Fukuoka, Japan; b Department of Anatomic Pathology, Pathological, Sciences, Graduate School of Medical Sciences, Kyushu University, Fukuoka, Japan; c Department of Neurosurgery, Clinical Research Institute, National Hospital Organization Kyushu Medical Center, Fukuoka, Japan; d Department of Neurosurgery, Juntendo University, Tokyo, Japan

**Keywords:** Oncology, Surgery, Cancer surgery, Neurosurgery, Ear-Nose-Throat, Jugular foramen, PEComa, Skull base tumor

## Abstract

**Background:**

Perivascular epithelioid cell tumors (PEComas) of the skull base are extremely rare. Here we report the first description of a malignant PEComa mimicking jugular foramen schwannoma and presenting as Collet-Sicard syndrome, and we review the previous literature on PEComas of the head, neck and skull base.

**Case description:**

A 29-year-old woman presented with hoarseness, dysphagia, vomiting, and headache. She was first diagnosed with Collet-Sicard syndrome caused by thrombosis of the sigmoid and transverse sinuses. She was treated with anticoagulant therapy, and the hoarseness and paralysis of the accessory nerve improved. Later, at age 31, the hoarseness again worsened. At another hospital, enhanced computed tomography revealed a tumor in the jugular foramen extending to the neck and medially displacing the internal carotid artery. She was referred to our hospital for further examination and was diagnosed with jugular foramen schwannoma causing thrombosis of the sinuses. At the one-year follow-up, the tumor had grown rapidly and had started to surround the internal carotid artery. We therefore performed a tissue biopsy of the tumor in the jugular foramen and neck. Based on pathological analysis, we made a definitive diagnosis of malignant PEComa.

**Conclusions:**

It may be extremely challenging to reach an accurate diagnosis of PEComa in the skull-base region, which can cause a delay in treatment initiation. When atypical clinical features for a skull-base tumor are found, we recommend preliminary biopsy to obtain a definitive diagnosis and initiate an appropriate treatment strategy as early as possible.

## 1 Introduction

In 1992, Bonetti et al. first described the concept of tumors involving perivascular epithelioid cells (PEC) as a distinct entity [1]. The name “PEComa” was later assigned to these tumors, by Zamboni in 1996 [2]. In 2002, the World Health Organization (WHO) recognized the concept of perivascular epithelioid cell tumors as a family of mesenchymal neoplasms [3], which includes angiomyolipoma, lymphangioleiomyomatosis, and clear cell “sugar” tumors. In general, PEComa is recognized as a benign tumor type, but it reportedly can have malignant characteristics.

The site of origin of this tumor is extremely variable; however, the head and neck are rare origin sites [3, 4, 5, 6, 7, 8, 9, 10, 11, 12, 13, 14, 15, 16, 17, 18, 19, 20, 21, 22, 23, 24, 25, 26, 27], and PEComas arising from the skull base are even more unusual [18, 23]. In this report, we describe a case with a PEComa located in the jugular foramen. In this region, paraganglioma and schwannoma are common tumors, and meningioma and metastatic tumors are also relatively frequent. Patients with these tumors may present with Collet-Sicard Syndrome, which is a rare condition characterized by unilateral palsy of the lower cranial nerves (CNs) IX, X, XI, and XII.

To our knowledge, we report here the first case of malignant PEComa arising from the jugular foramen. Interestingly, it mimicked jugular foramen schwannoma and resulted in Collet-Sicard syndrome. This made it extremely difficult to confirm the definitive diagnosis.

## 2 Case presentation

A 29-year-old woman without the history of oral contraceptives use presented with hoarseness, dysphagia, vomiting, and headache. Later physical examination in our neurology department also revealed paralysis of the right CNs IX to XII. Contrast-enhanced computed tomography (CT) of the head and neck revealed an interruption of flow in the right sigmoid and transverse sinuses, with no apparent tumor on contrast-enhanced CT scan. Coagulation profile including the prothrombin time, activated partial thromboplastin time was normal. She was diagnosed with Collet-Sicard syndrome caused by thrombosis of the sigmoid and transverse sinuses. She was treated with anticoagulant therapy, and the hoarseness and paralysis of the accessory nerve improved, but recanalization of the sigmoid sinus and transverse sinus was not identified. The hoarseness later worsened again, when she was 31 years old. In another hospital, contrast-enhanced CT scan revealed a tumor in the jugular foramen extending into the deep cervical region and medially displacing the internal carotid artery. The patient was referred to our hospital for further examination of this tumor. The tumor was investigated using contrast-enhanced CT and magnetic resonance imaging. It showed hypointensity on T1-weighted MRI and iso-to hyperintensity on T2-weighted MRI. The smooth tumor rim was enhanced on contrast-enhanced MRI. On a contrast-enhanced CT scan, the tumor displayed slightly heterogenous enhancement. She was diagnosed with jugular foramen schwannoma (Kaye's Type C) causing thrombosis of the sigmoid and transverse sinuses. No personal or family history of tuberous sclesosis was reported.

**Figure 1:**
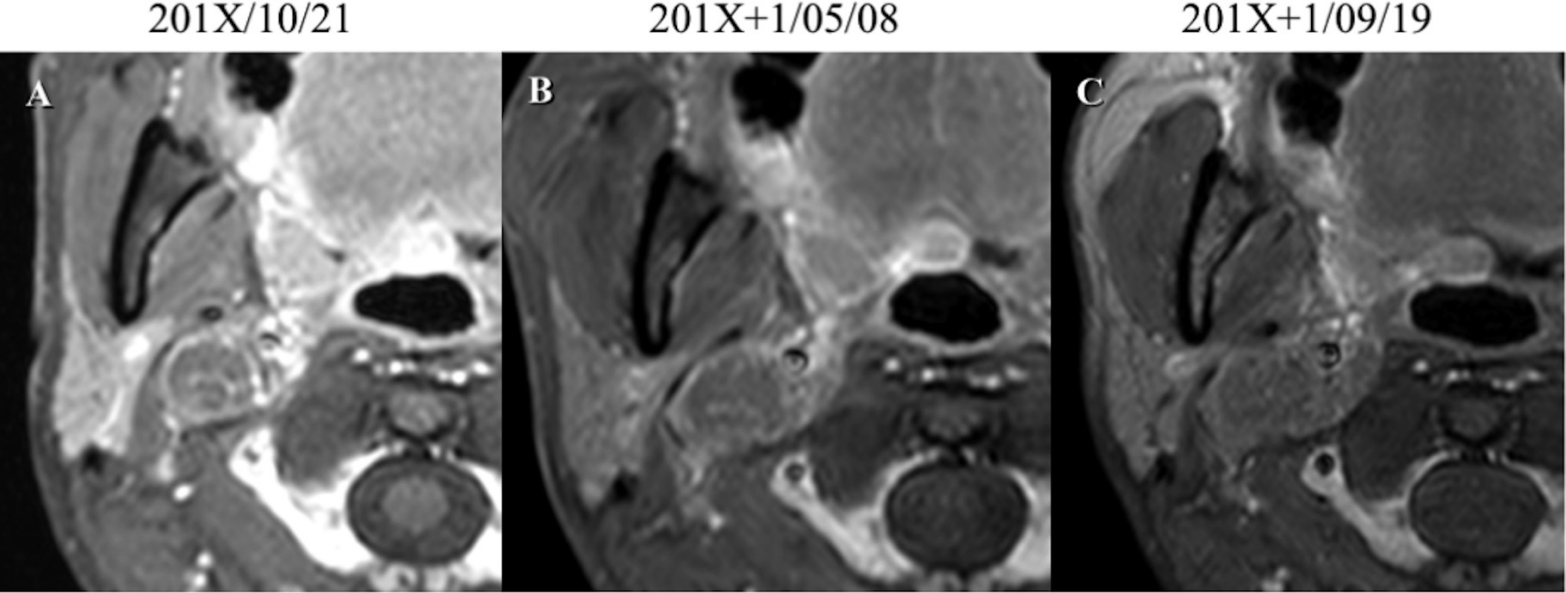
Tumor in the jugular foramen extending into the deep cervical region (Type C jugular foramen tumor). The tumor did not exhibit internal flow voids. It exhibited hypointensity on T1-weighted MRI, and iso-to hyperintensity on T2-weighted MRI. Furthermore, contrast-enhanced T1 MRI showed that the tumor rim was clearly enhanced (A). On a contrast-enhanced CT scan, this tumor displayed slightly heterogenous enhancement. Tumor had grown rapidly (B) and started to surround the internal carotid artery (C).

**Figure 2:**
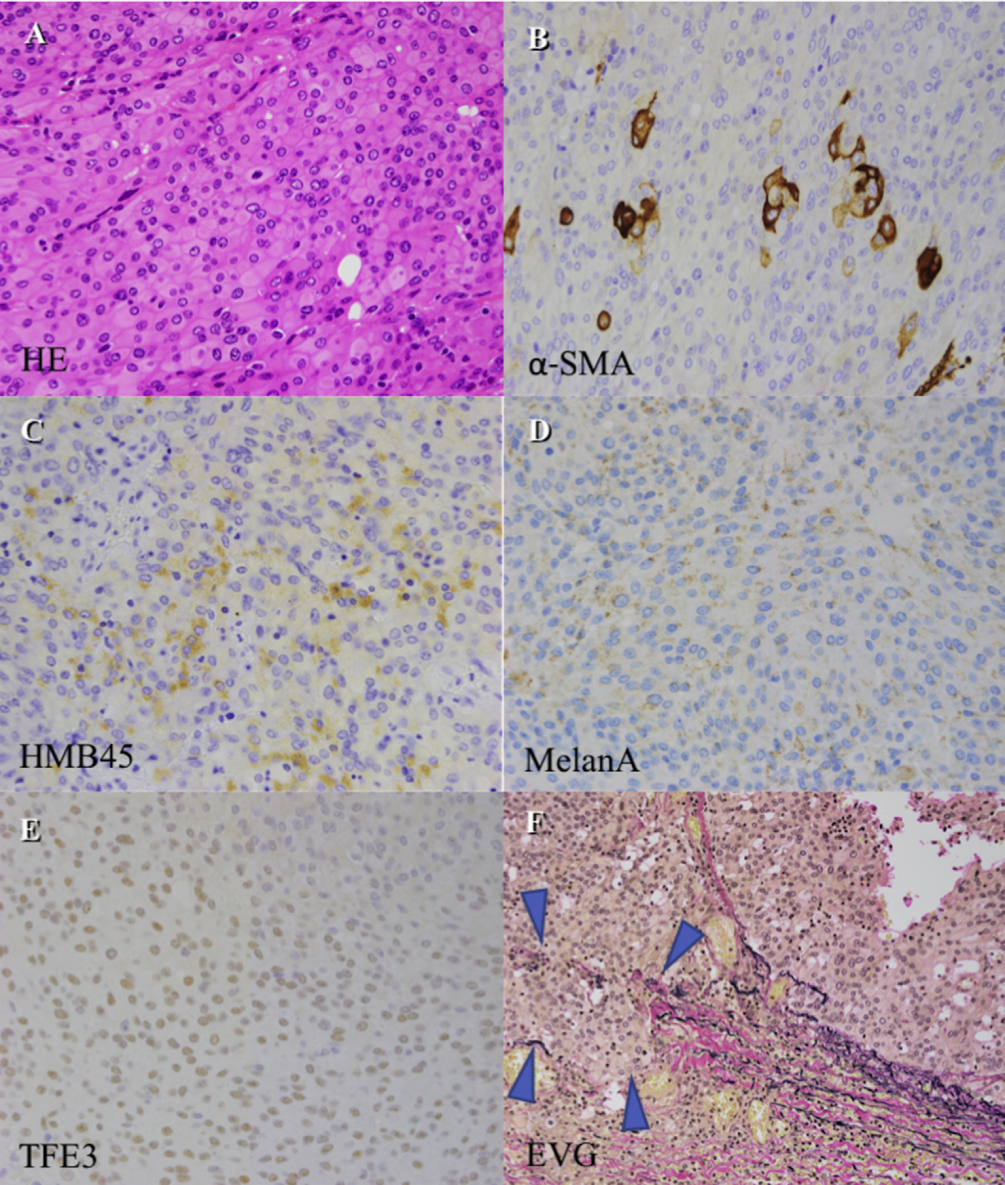
Histological sections revealed a proliferation of epithelioid cells with enlarged nuclei and eosinophilic or focally clear cytoplasm, arranged in sheets or nested patterns with narrow blood vessels (A). The mitotic index was 2/10 HPF. Necrosis was not evident. Immunohistochemically, tissue tumor cells were positive for α-SMA (B), HMB-45 (C), Melan-A (D), and TFE3 (E), but negative for desmin, AE1/AE3, S-100 protein,. The MIB-1 labeling index was approximately 5%. The blue arrows indicate vascular invasion highlighted by EVG staining (F).

**Figure 3:**
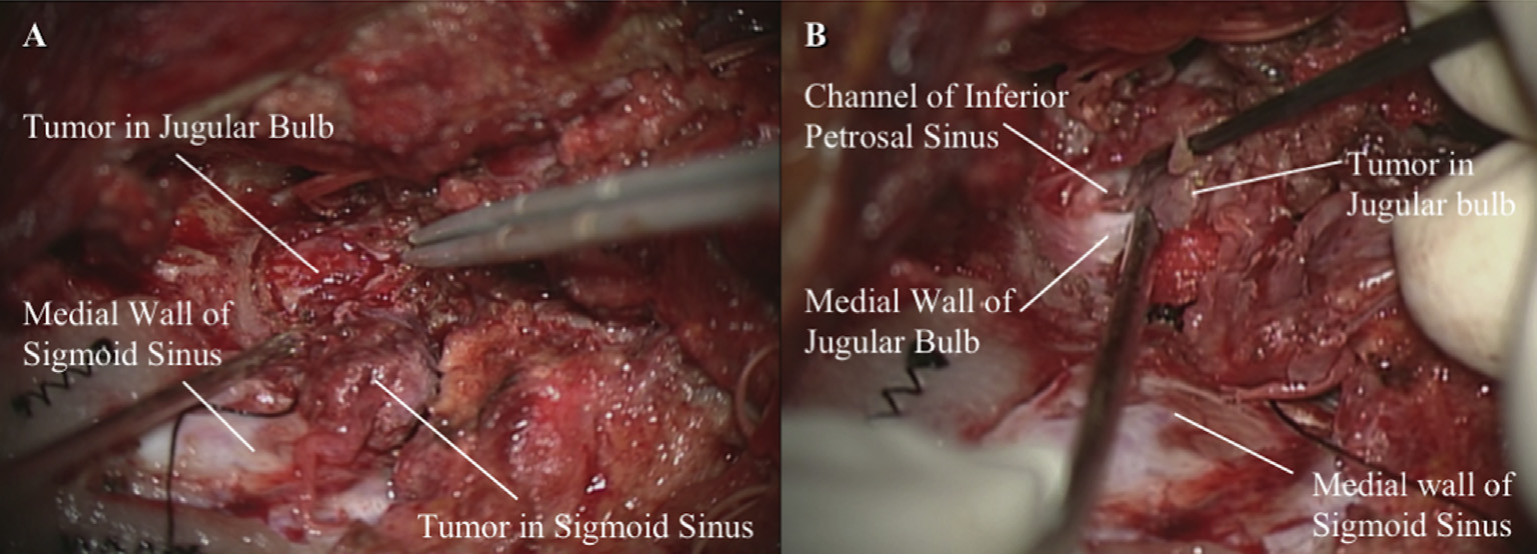
The tumor filled the sigmoid sinus and could be separated from the medial wall of the sigmoid sinus (A). It also filled the jugular bulb and extended into the inferior petrosal sinus and posterior condylar vein (B).

At the one-year follow-up, however, her tumor had grown rapidly, and we found that the tumor had started to surround the internal carotid artery (Figure 1). This clinical course was thought to be atypical for a jugular foramen schwannoma. We decided to perform a tissue biopsy of the tumor in both the jugular foramen and the deep cervical area. In open biopsy, the jugular bulb and internal jugular vein were filled with the tumor. Histological sections showed a proliferation of epithelioid cells with enlarged nuclei and eosinophilic or clear cytoplasm, arranged in sheets or nested patterns with narrow blood vessels. Immunohistochemically, the tissue tumor cells were positive for HMB-45, Melan-A, and α-smooth muscle actin (α-SMA), but negative for desmin, AE1/AE3, and S-100 protein. Pathological analysis revealed a perivascular epithelioid cell tumor. (Figure 2A tumor size of >5 cm with a mitotic index of 2 (counts per 10 high-power fields, HPF) and vascular invasion was identified. Based on these features, a diagnosis of PEComa with malignant potential was made. After discussing treatment options with otorhinolaryngologists, radiologists, and an oncologist, we decided to perform surgical treatment first. preoperatively, the superficial temporal artery-middle cerebral artery bypass was performed. We applied a postauricular transtemporal approach with anterior facial nerve rerouting to remove the tumor sacrificing the internal carotid artery involved by the tumor (Figure 3). However, it resulted in incomplete tumor resection, because the cardiac arrest and bradycardia was found during the removal of the tumor around the inferior petrosal sinus and anterior condylar confluence. The CNs IX to XI were sacrificed because these structures were invaded and it was extremely difficult to separate these structures from the tumor. Post-operative dysphagia, horseness and accessory nerve palsy were found. Six months after surgery, radiotherapy was added to treat the residual tumor volume. After one year follow up, there is no evidence of the regrowth of the residual tumor and metastasis.

Written informed consent for the current study was obtained before obtaining the patient's data.

## 3 Discussion

PEComas can arise in a variety of anatomic sites. According to our literature review, sites for head and neck PEComas include the nasal cavity [6, 7, 8, 12, 14, 21, 22, 25], scalp [5, 9, 10], neck soft tissue [10], eye [4, 11, 15, 16, 19, 24, 27], oral cavity [14, 20], larynx [6, 17], pharynx [17, 26], face [13], and skull base [18, 23]. PEComas arising from sites in the skull base like the jugular foramen are extremely rare. Our case is the first report of a jugular foramen PEComa mimicking a schwannoma and resulting in the Collet-Sicard syndrome.

Tumors arising from the jugular foramen vary from common tumors, including schwannomas, paragangliomas, meningiomas and metastatic tumors, to rare tumors such as chondrosarcoma and chordoma [28]. Jugular foramen schwannoma and paraganglioma are diagnosed based on the presence of characteristic radiographical features. However, there are no definitive imaging features used to diagnose PEComa, making it difficult to assign a definitive diagnosis without histological examination.

In our case, she was initially diagnosed with the Clollet-Sicard syndrome due to the thrombosis of the sigmoid and transverse sinuses, not tumorigenesis. It made the diagnosis process extremely confusing and difficult. As previously reported, the venous thrombosis around the jugular foramen can be cause of the lower cranial nerve paralysis. Several authors reported the paralysis of cranial nerves IX, X, XI, XII was caused by the thrombosis of the sigmoid-jugular complex similar to our case [29, 30, 31]. As well as thrombosis of the jugular foramen, jugular foramen shwannomas can present paralysis of CNs IX to XII. These findings made it difficult to deny the diagnosis of the schwannoma in our case. Because of the similarity in radiological findings between the tumor in this case and typical schwannomas, and previous reports on the cranial nerve paralysis caused by the schwannoma [32, 33, 34], our patient was diagnosed with jugular foramen schwannoma [32]. As a result, it was decided that appropriate approach for this case was to wait and scan. However, the growth pattern was atypical for a schwannoma, which led us to biopsy the tumor from the jugular foramen and deep cervical area.

PEComas are mainly composed of clear to eosinophilic epithelioid or spindle cells, or a mixture of both, which grew in nested and fascicular pattern [10]. Immunohistochemically, melanocytic markers, such as HMB-45 and Melan-A, and α-SMA are positive, but cytokeratin is typically negative [6, 10]. Although it has been recognized that PEComas do not express S-100 protein, some studies indicate positivity of S-100 protein [10, 17, 21]. Co-expression of melanocytic markers and SMA is considered to be the hallmark of PEComa. Our case met these histopathological and immunohistorical features, and ultimately a diagnosis of PEComa was made. Traditionally, PEComas were thought to be benign tumors, but malignant disease courses with local recurrence and distant metastases have been reported [4, 8, 9, 10, 14, 17, 18, 23, 26]. Folpe et al. proposed that tumors with two or more of the following defining features are considered malignant: tumor size >5 cm, infiltrative growth pattern, high nuclear grade and cellularity, mitotic rate ≥1/50 HPF, necrosis, and vascular invasion [10]. Tumors composed of nuclear pleomorphism or multinucleated giant cells only, or with a size >5 cm, are considered to be of uncertain malignant potential. The tumor in our case showed infiltrative growth, vascular invasion, nuclear atypia, and high mitotic activity (2/10 HPF), and was thus considered to have malignant potential. However, the application of these criteria to all PEComas arising from head and neck sites remains controversial. Based on Folpe's criteria, 12 out of 31 previously reported cases were considered to have malignant potential (Table 1). Two cases of PEComa arising from the skull base have been reported, and both had malignant potential [18, 23]. One of them resulted in lung and spine metastasis, and the patient died from the disease [23].

**Table 1:** Perivascular epithelioid cell tumor of the head and neck region. Asterisks indicate the cases with malignant potential, based on Folpe's criteria.

Case	Year	Author	Size (cm)	Location	Sex	Age	Treatment	Histologic Features	Infiltrative Growth	Vascular Invasion	Nuclear Atypia	Mitoses/10HPF	Necrosis	Follow-up	Immunohistochemical Features					
															HMB-45	Melan-A/MART-1	S-100	SMA	desmin	TFE3
1	2001	SS. Banerjee	2	Nasal Cavity	F	39	SR	Mixed	NA	NA	-	Very few	-	12 months/ANED	+	Focally	-	+	+	NA
2*	2004	NL. Lehman	5	Skull Base	F	49	Not Treated	Epi.	+	+	-	3 per HPF	-	Spine, Lung met, DOD	+	Few	-	+	-	NA
3*	2005	AL.Folpe	2	Scalp	M	80	NA	Epi.	NA	+	+	>10 per 50 HPF	-	Lost to follow -up	NA	NA	NA	NA	NA	NA
4*	2005		2.6	Neck Soft Tissue	F	77	SR→ RT	Mixed	NA	-	+	1 per 50 HPF	-	6 months/ANED	NA	NA	NA	NA	NA	NA
5	2005	P. Iyengar	1.2	Orbit	F	9	SR	Epi.	NA	-	-	Very few	-	7 months/ANED	+	-	-	+	-	NA
6	2005	IG. Koutlas	4	Hard Palate	F	46	SR	Mixed	-	-	+	-	-	20 months/ANED	+	+	-	+	+	NA
7	2008	R. Guthoff	1.5	Orbit	M	54	SR	Epi.	NA	NA	-	Very few	-	17 months/ANED	+	+	-	NA	-	NA
8*	2008	KB. Calder	1.6	Scalp	M	76	SR	Epi.	-	-	+	2 per 10HPF	-	Cervical node met (5years previously)	+	+	-	+	NA	NA
9	2009	N. Kuroda	N/A	Nasal Cavity	M	79	NA	Epi.	NA	NA	-	-	-	Follow up too short	+	NA	+	+	NA	+
10*	2009	S. Huai-yin	5	Vocal Cord	F	38	SR	Epi.	+	NA	no or only mild atypia with low mitotic activity, two cases focally showed moderate atypia and mitotic activity 1–2/10HPF		-	Recur at 15 months→Second ope	+	+	+	+	NA	NA
11	2009		3.5	laryngeal Vestibule	M	42	SR	Mixed	-	NA			-	13 months/ANED	+	+	-	+	NA	NA
12	2009		3	Hypopharynx	M	47	SR	Epi.	-	NA			-	30 months/ANED	+	+	-	+	NA	NA
13	2009	Panelos	N/A	Nasal Septum	F	50	SR	Mixed	-	NA	-	Very few	-	6 years/ANED	+	-	-	+	+	NA
14	2010	E. Furusato	2	Upper Eyelid	F	26	SR	Epi.	+	NA	-	Very few	-	24 months/ANED	+	+	-	+	+	+
15	2010		1.33	Inferior Ciliary body	M	7	SR	Epi.	+	NA	-	Very few	-	24 months/ANED	+	-	-	+	+	+
16	2010	N. Ghazali	2cm	Cheek	F	32	SR	Mixed	-	-	-	2 per HPF	-	4 years/ANED	-	+	-	+	+	NA
17	2010	P. Argani	2cm	Scalp	M	80	NA	Epi.	NA	NA	+	-	-	NA	+	-	-	NA	+	-
18	2011	A. Bandhlish	2.9	Nasal Cavity	F	18	SR	Epi.	NA	NR	-	-	-	26 months/ANED	+	+	-	-	-	NA
19	2011		N/A	Nasal Cavity	F	71	NA	Epi.	NA	NR	-	-	-	Lost to follow-up	+	NA	-	+	-	NA
20	2011		N/A	Glottis	F	26	SR	Epi.	NA	NA	-	-	-	8 years/ANED	+	NA	-	+	NA	NA
21	2012	B. Leavers	4	Maxillary Nasal Process	F	74	SR	Epi.	+	NA	-	-	-	12 month/ANED	+		NA	NA	NA	NA
22	2012	S. Gana	1.5	Nasal Cavity	F	22	SR	Mixed	NA	NA	+	-	-	13 months/ANED	+	+	-	+	+	-
23*	2013	C. Bocciolini	N/A	Nasal Cavity	F	40	SR	Epi.	+	NA	+	5/50HPF	+	7 years/ANED	+	+	-	-	-	NA
24	2014	H. Goto	1.1	Ciliary Body	F	13	SR	Epi.	NA	NA	-	-	-	4 years/ANED	+	+	-	+	-	+
25	2016	I. Lubo	2	Intraorbital	M	47	SR	Epi.	-	NA	-	2/10HPF	-	40 months/ANED	+	-	-	+	-	NA
26*	2017	K. Saluja	7.2	Oropharynx	F	28	mTOR-I→RT→SR	Epi.	NA	-	+	Up to 6/10HPF	+	6 month	+	-	-	+	+	+
27	2017	A. Varan	3.4	Orbit	M	7	Chemo.	Epi.	NA	NA	-	-	+	6 month/tumor size stable	-	+	NA	+	+	+
28*	2017	MS. Alam	>5	Orbit	M	5	Chem.→SR→Chemo.	Epi.	NA	NA	+	+	NR	24 months/ANED	+		-	+	NA	NA
29*	2017	MD. Hyrcza	2.3	Sella Turcica	F	45	SR	Epi.	NA	NA	+	Up tp 1/10HPF	-	NA	+	NA	-	Focally	-	+
30*	2017	Goodman	3.5	Base of Tongue	M	55	SR	Epi.	NA	NA	+	≥1/50HPF	-	5 months/ANED	-	+	-	-	NA	NA
31*	2017		3	Sinus	F	48	SR	Epi.	NA	NA	+	≥1/50HPF	-	17 monthes/ANED	+	+	NA	+	NA	NA
32	2017		1.2	Anterior Nasal	M	8	SR	Epi.	NA	NA	-	-	-	NA	-	-	NA	+	NA	NA
33*	2018	N. Komune	6.7	Jugular Foramen	F	31	SR→ RT	Epi.	+	+	+	2/10HPF	-		+	+	-	+	-	+

ANED, alive with no evidence of disease; Chemo., chemotherapy; Epi., epithelial; HPF, high-power field; Met., metastasis; mTOR, mammalian target of rapamycin inhibitor; NA, not available; Reccur., recurrence; RT, radiation therapy; SR, surgical resection.

During the open biopsy on our patient, the tumor was found to be mainly within the jugular bulb and internal jugular vein. This was one of the reasons for its smooth round shape, which made it look similar to jugular foramen schwannoma. Its infiltration into the sigmoid sinus was thought to be the cause of the thrombosis of the sigmoid and transverse sinuses, and it extended into the internal jugular vein, where it acquired the radiological appearance of a schwannoma.

The optimal treatment strategy for perivascular epithelioid cell tumors remains controversial, but surgical resection with adequate surgical margins is thought to offer the best prognosis. Even if the tumor has malignant potential, surgical treatment offers local control, and our literature review supports surgical intervention as the first choice for both benign or malignant PEComas of the head and neck (Table 1). It is not clear whether radiotherapy and chemotherapy are unequivocally indicated, but they may be for tumors with malignant potential or in cases with incomplete resection. For cases of PEComas in the head and neck region, three groups have reported on neoadjuvant or/and adjuvant therapy combined with surgery in the treatment of malignant cases. In all three cases, the patients remained alive for 6–24 months after treatment, with no evidence of recurrence [4, 10, 26].

Although the use of mTOR inhibitor therapy remains controversial, mTOR inhibitors have been considered as an option for treatment, based on the involvement of mTOR pathway activation [26], and because conventional PEComa frequently harbor mutation and loss of heterozygosity of tuberous sclerosis complex (TSC) 2 and much more rarely, TSC1. The significance of loss of heterozygosity at TSC1/2 play a role in regulation the rapamycin (mTOR) pathway [35, 36]. Recently, transcription factor E3 (TFE3) rearranged tumors were shown to lack TSC2 inactivating mutations [37]. the rearranged variant of PEComa may be less responsive to mTOR inhibitor [36]. Saluja et al. reported a malignant oropharynx PEComa with strong TFE3 protein expression and gene rearrangement. This case was treated with the mTOR inhibitor everolimus for a month, but this treatment was unsuccessful [26], so a combination of adriamycin and ifosfamide was used for a month. A palliative dose of radiotherapy to the neck was then added, followed by surgery [26]. Six months after surgery, no local recurrence or distant metastasis were noted. Thusly, the most reliable treatment is still surgical removal. It is therefore essential to make a definitive diagnosis of this type of tumor based on pathological findings. If total removal of tumor cant not be achieved, radiation or adjuvant chemotherapy should be considered soon after histopathological examination result is reported.

In summary, it may be extremely challenging to reach an accurate diagnosis of atypical tumors in the skull-base region, which may result in a delay in treatment initiation. The radiological findings in this case were extremely similar to those for jugular foramen schwannoma; however, the clinical course was not typical of schwannoma. Even if a case has radiographical similarity to common tumors, and despite the fact that biopsy of the jugular foramen tumor is often challenging, a biopsy should be considered in cases with atypical clinical courses and features, in order to obtain a definitive diagnosis and initiate an appropriate treatment strategy as early as possible.

## Declarations

### Author contribution statement

All authors listed have significantly contributed to the investigation, development and writing of this article.

### Funding statement

This work was supported by JSPS KAKENHI Grant Number JP 18K16895.

### Competing interest statement

The authors declare no conflict of interest.

### Additional information

No additional information is available for this paper.
